# Introductory Article on Global Burden and Epidemiology of Typhoid Fever

**DOI:** 10.4269/ajtmh.18-0032

**Published:** 2018-07-25

**Authors:** Amruta Radhakrishnan, Daina Als, Eric D. Mintz, John A. Crump, Jefferey Stanaway, Robert F. Breiman, Zulfiqar A. Bhutta

**Affiliations:** 1 Centre for Global Child Health, The Hospital for Sick Children, Toronto, Canada;; 2 National Center for Emerging and Zoonotic Infectious Diseases, Centers for Disease Control and Prevention, Atlanta, Georgia;; 3 Centre for International Health, University of Otago, Dunedin, New Zealand;; 4 Institute for Health Metrics and Evaluation, University of Washington, Seattle, Washington;; 5 Emory University, Atlanta, Georgia;; 6 Dalla Lana School of Public Health, University of Toronto, Toronto, Canada;; 7 Center of Excellence in Women and Child Health, The Aga Khan University, Karachi, Pakistan

## Abstract

This article is the introduction to a 12-paper supplement on global trends in typhoid fever. The Tackling Typhoid (T2) project was initiated in 2015 to synthesize the existing body of literature on typhoidal *salmonellae* and study national and regional typhoid fever trends. In addition to a global systematic review, eight case studies were undertaken to examine typhoid and paratyphoid fever trends in endemic countries alongside changes in relevant contextual factors. Incidence variations exist both within and between regions with large subnational differences as well, suggesting that public health changes impacting typhoid and paratyphoid fevers in one setting may not have similar impacts in another. This supplement also brings to light the lack of national typhoid fever surveillance systems, inconsistencies in diagnostics, and the lack of typhoid fever associated morbidity and mortality data in many countries, making it difficult to accurately quantify and track burden of disease. To better understand typhoid fever there is a need for more high-quality data from resource-poor settings. The implementation of typhoid surveillance systems alongside the transition to blood-culture confirmation of cases, where possible, would aid in the improvement of data quality in low-income settings. The following supplement includes the results of our global systematic review, eight-country case study articles, a qualitative article informed by semistructured interviews, and a conclusion article on potential ways forward for typhoid control.

## INTRODUCTION

This article introduces a collection of studies undertaken within the “Tackling Typhoid” (T2) project, which aimed to consolidate the current body of literature on national and regional typhoid fever trends in incidence, mortality, and severe complications. The T2 project included a systematic review of the literature and eight-country case studies in Chile, Nigeria, South Africa, Pakistan, India, Bangladesh, Thailand, and Vietnam. Case studies examined typhoid and paratyphoid fever incidence trends in conjunction with changes in relevant contextual factors, such as water treatment and distribution, sanitation infrastructure, female literacy, poverty rates, and diarrheal mortality, and included in-depth interviews with local public health experts to identify local interventions and control measures that were implemented to reduce the transmission of typhoid fever directly or indirectly by targeting other infectious disease. The studies in this supplement collectively characterize global and regional trends in typhoid and paratyphoid fever, and the findings can inform recommendations on interventions and policies that can best help curb the continued spread of this disease.

### Epidemiology.

**Figure 1.**
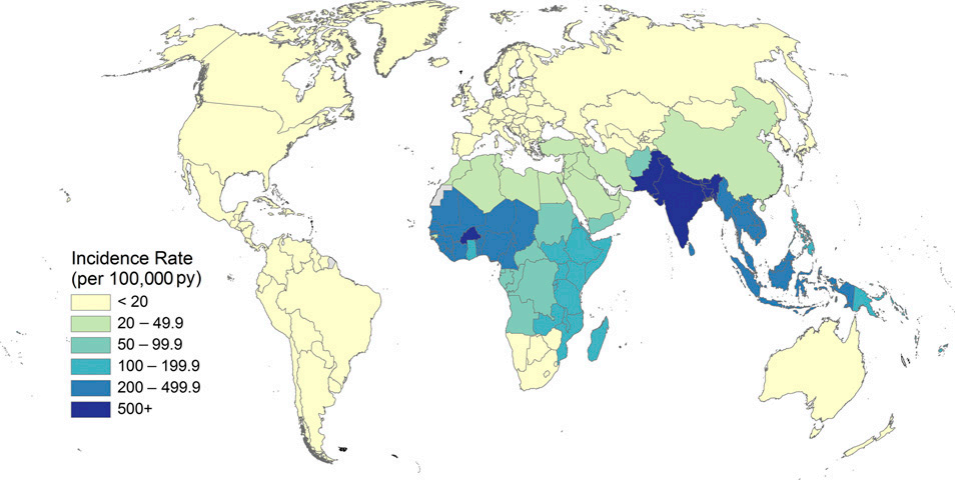
Estimated incidence of typhoid and paratyphoid fevers by country per 100,000 population, 2015.

Typhoid and paratyphoid fever are enteric infections caused by the bacteria *Salmonella enterica* serovar Typhi (*S.* Typhi) and Paratyphi A, B, and C (*S.* Paratyphi A, B, and C), respectively, collectively referred to as typhoidal *Salmonella*, and the causes of enteric fever.^1^ Humans are the only reservoir for *Salmonella* Typhi with disease transmission occurring via the fecal–oral route, usually through the consumption of food or water contaminated by human feces.^2^ An estimated 17 million cases of typhoid and paratyphoid fever illnesses occurred globally in 2015,^3^ mostly in South Asia, Southeast Asia, and sub-Saharan Africa, with both the largest burden and incidence occurring in South Asia (Figure 1).^3,4^ Left untreated, both typhoid and paratyphoid fever may be fatal^1^ with 178,000 deaths estimated worldwide in 2015.^3^

Although considerable literature exists on typhoid fever incidence, most endemic countries do not have well-established population-based national surveillance systems for typhoid fever. In addition, some countries that use passive surveillance use clinical diagnoses with limited ability to confirm typhoid fever cases by blood culture. Most data are, therefore, collected from hospital-based studies, leaving substantial knowledge gaps in certain geographies, especially where health-care usage is low. A review of global burden^5^ showed that from 1954 to 2000 only 13 countries had population-based surveillance data for typhoid fever, only two of which were in Africa—Egypt and South Africa. At the time, both these countries had surveillance data from the control arms of vaccination trials only,^5^ although considerably more data have become available since. Data from Ministry of Health surveillance reports in Thailand highlight a shift from *S.* Typhi as the primary typhoidal *Salmonella* bacteria isolated to *S.* Paratyphi.^6^ Thailand is broken down into seven regions, of which four are showing this transition between 2004 and 2014.^6–8^ Within the Bangkok and Vicinities region, two provinces (Bangkok and Samut Prakan) of the six that comprise the region show *S.* Typhi incidence decreasing as *S.* Paratyphi increases.^6,7^ This shift is also observed in three provinces (Ratchaburi, Kanchanaburi, and Phetchaburi) from the western region of Thailand.^6,7^ Although improvements in water, sanitation infrastructure, and public health measures have led to the virtual disappearance of typhoid fever transmission within the developed world, residual cases largely occur in travelers returning from countries where typhoid fever remains endemic.^9^ Knowledge of local disease burden, risk factors for acquisition, transmission characteristics, and implemented control measures are essential in developing strategies for prioritized and optimally targeted typhoid and paratyphoid fever control, and elimination.

#### Morbidity.

Typhoid fever incidence has decreased to very low levels in developed countries such as the United States and Canada.^9^ In 1990, surveillance from the United States recorded incidence at 0.22 cases per 100,000 persons per year. Incidence has since decreased even further and remained consistently below 0.16 cases per 100,000 persons per year as of 1995.^10^ A study assessed 1,872 cases of typhoid fever from 2008 to 2012 of which 86% were associated with foreign travel. The small number of cases still being acquired domestically demonstrates that typhoid fever remains a constant, albeit minor problem within the United States.^9^

Although most cases of typhoid fever occur in Asia and Africa, considerable regional differences exist, both within and between countries. Data from the Diseases of the Most Impoverished (DOMI) population-based surveillance study, led by the International Vaccine Institute (Seoul, South Korea), estimated the overall incidence of 493.5 cases per 100,000 person-years in children aged 5–15 years in an urban slum in Kolkata, India (2003–2004).^11^ A similar population-based study conducted in the Dong Thap Province in the south of Vietnam from December 1995 to December 1996 estimated typhoid fever incidence at 198 per 100,000 person-years.^12^ A comparable incidence was observed in Dhaka between January 2003 and January 2004 where typhoid fever incidence was estimated to be 200 cases per 100,000 person-years.^13^ Most previous typhoid fever estimates from sub-Saharan African countries were from hospital-based studies,^14^ although earlier population-based studies from Kenya did suggest high rates in an urban slum of 247 cases per 100,000 person-years observed between 2007 and 2009. This study also showed 15-fold higher incidence in urban children compared with their rural counterparts.^15^ A recent publication describes population-based studies of typhoid fever and invasive nontyphoidal *Salmonella* disease in 12 sites in 10 countries across sub-Saharan Africa.^16^ Incidence estimates ranged from 0 to 383 cases per 100,000 person-years across the 12 sites. Although poor infrastructure and unstable governments make the establishment of robust disease surveillance systems difficult in resource-constrained settings,^17^ these new data from sub-Saharan Africa will help in forming more accurate future estimations of the global burden.

#### Age-specific typhoid incidence.

Typhoid fever incidence varies by age. In endemic countries, the highest incidence is in younger children, whereas incidence is similar in all age groups in low-burden settings. A study from 2004 used data from published studies to extrapolate incidence rates by age group and reported the highest incidence in children under the age of 5 years in high incidence settings.^5^ Modeled estimates from the 2015 Global Burden of Disease study (GBD 2015) showed typhoid fever incidence rates decreasing as age increased.^3^ Furthermore, results from the DOMI study conducted in five endemic countries demonstrate substantial heterogeneity in typhoid fever incidence across age groups. The heterogeneity across age groups was observed in all DOMI study sites and sites from the Typhoid Fever Surveillance in Africa Program.^11,16^

#### Mortality.

**Figure 2.**
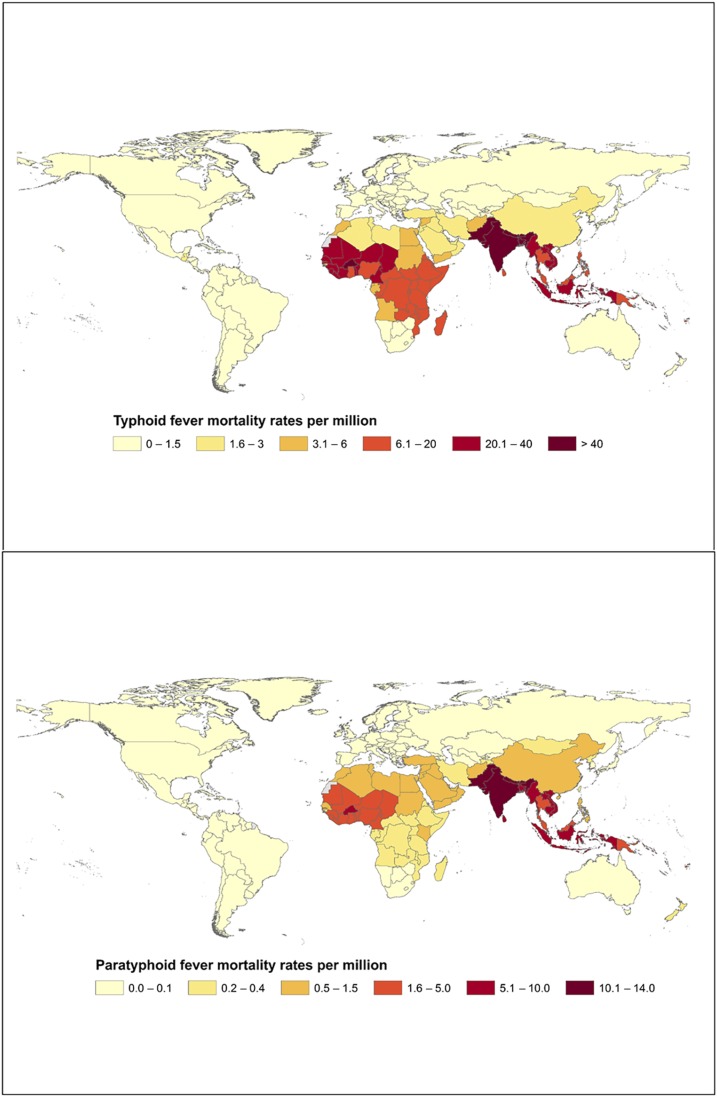
Estimated global mortality from typhoid and paratyphoid fever by country per million, 2015.

Mortality from typhoid and paratyphoid fevers is difficult to estimate, because cases identified with typhoid fever during surveillance should receive appropriate clinical management and deaths presumed due to typhoid should receive scrutiny to rule out other possible causes. Nonetheless, typhoid and paratyphoid fevers were estimated to be associated with approximately 200,000 deaths in 2000 with absolute number of deaths estimated to be the highest in Africa and South Asia.^5^ Figure 2 presents global typhoid and paratyphoid mortality estimates from GBD 2015. Deaths due to paratyphoid fever tend to be lower than those of typhoid fever.^18^ However, this difference could be attributed to the incidence of paratyphoid fever being lower than that of typhoid in many settings. The number of paratyphoid fever deaths is estimated to be the greatest in South Asia, where its incidence is also the greatest although far lower than that of typhoid. Before the introduction of antimicrobials, death occurred in as many as 33% of typhoid fever patients in hospital and community settings from developing countries and was seen in upward of 10% of cases in developed countries.^19^ The use of antimicrobials initially lowered typhoid fever case fatality below 2%^19^ but the emergence of antimicrobial resistant strains in high-burden countries has been a growing concern in recent years.

#### Strategies for typhoid control and preventive measures.

A range of strategies exist to prevent and control typhoid fever (Table 1). Factors such as crowding, poor sanitation, unsafe water, and unsafe food production and handling processes contribute to *Salmonella* Typhi and *Salmonella* Paratyphi transmission. Therefore, measures to interrupt transmission through improvements in sanitation, drinking water, and food production need to be included in comprehensive prevention strategies for enteric fever.^10^ In addition to the aforementioned strategies that target risk factors, interventions focused on timely diagnosis and appropriate clinical management can also improve typhoid fever outcomes (Table 1).

**Table 1:** Control measures for the management of typhoidal *Salmonella*

Level	Interventions
Water and sanitation infrastructure	Ready access to potable water
	Use of improved sanitation
	Sewage collection and treatment
Health systems	Accurate, rapid diagnosis, and antimicrobial susceptibility testing
	Identification and treatment of chronic carriers
	Appropriate antimicrobial treatment
	Vaccination*
	Food safety regulations, implementation, and enforcement
Education	Handwashing before eating and before food preparation and after defecation^20^
	Food safety education

* Vaccines for paratyphoid fever are not available.

The use of vaccines in the control of typhoid fever has been successful as a preventative measure and during outbreak situations in many contexts. In China, during the 1999 typhoid outbreak, the Vi capsular polysaccharide (ViCPS) vaccine showed a protective efficacy of 73% in children previously vaccinated and 71% in children who received the vaccine during the outbreak.^21^ In Thailand in 1977 a national typhoid immunization program was implemented in schoolchildren using the heat/phenol-inactivated typhoid vaccine. After the introduction of this program the isolation rate of *S.* Typhi decreased from 4.6% in 1976 to 0.3% in 1985.^22^

#### Early diagnosis of typhoid and paratyphoid fever.

From a clinician’s perspective typhoid and paratyphoid fever are indistinguishable. Furthermore, many other acute febrile illnesses such as dengue, leptospirosis, and malaria may present a clinical picture similar to that of typhoid fever. Accurate diagnosis requires laboratory confirmation.^23^ The development of practical, affordable, and accurate (i.e., both sensitive and specific) diagnostic tools is key to typhoid fever management and control. Typhoid fever can be diagnosed using a number of methods including culture of blood, bone marrow, or stool, and nucleic acid amplification tests (NAAT) for detecting bacterial nucleic acids in appropriate body fluids including blood or bone marrow.^24^ Techniques such as NAAT and bone marrow cultures are not, however, feasible in low-income settings, and blood culture or Widal tests are more commonly used.

Not all diagnostic methods perform equally well. Bacterial culture with bone marrow offers the greatest sensitivity at upward of 80%.^23^ However, bone marrow aspiration and culture is expensive and invasive and is not commonly used in practice. Consequently, although less sensitive, blood culture remains the practical standard for typhoid fever diagnosis.^23^ A recent systematic review of 10 studies examined the sensitivity of blood cultures relative to bone marrow cultures from a sample of 529 positive *Salmonella* Typhi cases (using blood and bone marrow positivity as a composite measure) and found that bone marrow cultures were positive in 96% of typhoid fever cases whereas blood cultures were positive in 61%.^25^ A study by Lee et al.^26^ states to accurately detect bloodstream infections such as *S.* Typhi, two to three 20 mL samples are required for adults. Blood culture sensitivity is the greatest in the initial stages of the infection and, with an adequate sample, has been observed to approach the sensitivity of bone marrow culture.^24,26,27^ Still, relying on blood cultures alone will underestimate the true incidence of typhoid fever. Stool cultures are still used in many endemic regions. However, a positive result may occur in acute disease, convalescent shedding, or chronic carriage.^28^ The Widal agglutination test works by measuring antibodies in serum. Although still used in resource-poor settings, the Widal test is inferior to blood culture in terms of both sensitivity and specificity.^28^ Other serological detection tests, although sometimes superior to Widal, also lack accuracy for typhoid fever diagnosis.^23^

Because sensitivity is < 100%, and has the potential to be influenced by over- or underfill of sample vials and contaminants within samples, case detection with a single-blood culture underestimates typhoid fever incidence. Antimicrobial use, which is a particular concern in high-burden regions, can inhibit bacterial growth and produce false-negative blood cultures, also resulting in underestimating typhoid fever prevalence in febrile patients. Additional diagnostic challenges in low-resource settings include capacity limitations (i.e., shortages in trained staff, diagnostic tools, quality control etc.) that make it difficult to distinguish typhoid from paratyphoid fevers and other causes of acute febrile illness.^29^

#### Treatment with antimicrobial agents.

Effective treatment with antimicrobials was introduced in the 1950s and has been shown to decrease typhoid fever case fatality risk from 30% to 0.5%.^30^ However, multidrug-resistant (MDR) typhoidal *Salmonella*, defined as strains resistant to ampicillin, chloramphenicol, and trimethoprim sulfamethoxazole, emerged in the 1980s. This led to the adoption of fluoroquinolones, such as ciprofloxacin, as the new first-line treatment with extended-spectrum cephalosporins as an alternative.^31^ *Salmonella* strains with decreased susceptibility to ciprofloxacin and cephalosporins have also begun to emerge in high-burden regions, such as South and Southeast Asia.^32^ As resistance to standard antimicrobial classes increase, new antimicrobials such as carbapenems, tigecycline, and azithromycin are being assessed as potential treatment options.^33^ The transition to new antimicrobials for treatment has caused a drop in MDR-resistant strains of *Salmonella* Typhi.^34^

#### Vaccines.

Three typhoid fever vaccines are currently pre-qualified by the World Health Organization and licensed for use in many countries, the Ty21a oral vaccine, the ViCPS injectable vaccine and more recently the first conjugate vaccine Tybar typhoid conjugate vaccine (TCV), already in use in India and Nepal in babies as young as 6 months of age.^35,36^ The Ty21a vaccine contains a live, attenuated strain of *Salmonella* Typhi and demonstrates a protective efficacy between 67% and 80% and the ViCPS vaccine provides a 72% protective efficacy when used in endemic areas and during an outbreak situation.^37−39^ A trial conducted using the new Tybar TCV vaccine showed the protective vaccine efficacy to be 54.6%.^36^ Of the two older vaccines, neither is licensed for use in children less than 2 years of age^35^ and while providing protective benefits against typhoid fever neither protects against infection with *Salmonella* Paratyphi for which no vaccine is currently available.

## CONCLUSION

Despite more information of higher quality available now than ever before, and a range of proven options for prevention, gaps remain in our understanding of typhoid fever burden and the best way to implement prevention strategies in low-resource settings. The findings from our comprehensive exercise to characterize trends in typhoid fever both globally and within endemic countries, summarized in this supplement, will help to fill some of these remaining gaps. The decisions to implement appropriate public health measures and preventive strategies for typhoid fever and other invasive *Salmonella* infections depend, in part, on the availability of locally improved laboratory diagnostics and relevant data on burden and contextual factors. Accurately estimating the burden of typhoid fever is challenging because data are scarce and derived from varied methods.^1^ There is uncertainty around the relative value of investments in health systems and large-scale engineering interventions, such as investments in water and sanitation, food safety measures, public awareness, improved diagnostics, treatment strategies, and immunization programs. The heterogeneity present in water, sanitation, and hygiene infrastructure, sociodemographic determinants, diagnostic test methods, and therapeutic procedures both within and between countries with endemic typhoid fever suggests that enteric fever cannot be eliminated by a single solution in every setting. There is hope regarding the control of enteric fever with the availability of the new Tybar TCV conjugate vaccine used as a complementary tool with the usual public health recommendations on water supply and sanitation.
